# Statin Treatment in the Acute Phase and the Risk of Post-stroke Pneumonia: A Retrospective Cohort Study

**DOI:** 10.3389/fneur.2021.635079

**Published:** 2021-09-06

**Authors:** Changling Li, Mengmeng Ma, Shuju Dong, Ye Hong, Jiajia Bao, Yang Zhang, Lijie Gao, Chaohua Cui, Jian Guo, Li He

**Affiliations:** Department of Neurology, West China Hospital of Sichuan University, Chengdu, China

**Keywords:** statin, post-stroke infection, post-stroke pneumonia, risk, severity

## Abstract

**Background:** We aimed to investigate the impact of statin treatment in the acute phase on the risk and severity of post-stroke pneumonia because of the uncertain effects of statins on post-stroke pneumonia.

**Methods:** Consecutive cases of acute ischemic stroke (AIS) between January 2014 and February 2019 were retrospectively analyzed. Additionally, the association of statin treatment in the acute phase with the risk and severity of post-stroke pneumonia was estimated with logistic regression. We registered the present study in the Chinese Clinical Trial Registry (ChiCTR 2000032838).

**Results:** Of the 1,258 enrolled patients, no significant difference was observed in post-stroke pneumonia risk between the two groups (with/without statin treatment in the acute phase) after propensity score matching (35.1 *vs*. 27.9%, *p* = 0.155). We did not find statin treatment in the acute phase to significantly increase the risk of post-stroke pneumonia both before and after matched analysis [odds ratio (OR) = 1.51, 95% confidence interval (CI) = 0.85–2.67, *p* = 0.157; OR = 1.57, 95% CI = 0.77–3.18, *p* = 0.213, respectively]. In the 271 patients with post-stroke pneumonia, no significant difference was found in its severity between two groups (19.6 *vs*. 19.4%, *p* = 0.964). No significant association was found between statin treatment and post-stroke pneumonia severity (OR = 0.95, 95% CI = 0.39–2.31, *p* = 0.918).

**Conclusions:** There appeared to be no additional benefits of statin treatment in the acute phase for post-stroke pneumonia reduction among AIS patients.

**Clinical Trial Registration:**http://www.chictr.org.cn, identifier: ChiCTR2000032838.

## Introduction

Infectious complications are common and could influence up to 65% of acute ischemic stroke (AIS) patients (1). Post-stroke infections (PSI) are defined as infections that occurred 48 h after the stroke and were not infected or in the latent period of infection at the time of onset (2, 3). Post-stroke pneumonia is a more common type of PSI (4), and ~75% of post-stroke pneumonia occur within the first 72 h of hospitalization (5). In addition, post-stroke pneumonia correlates with a third of early deaths and a fifth of poor outcomes in stroke (4, 6).

Apart from its role in cholesterol reduction, statins have anti-inflammatory, immunomodulatory, antioxidant, and endothelium-stabilizing effects, to name a few (7–10). Several observational studies have shown the effect of early statin use on reducing the infection risk in non-stroke patient populations (11–14). In addition to primary prevention, statins are recommended for secondary prevention of AIS (15, 16). However, the role of statins in post-stroke pneumonia risk is debatable. Whether statin treatment prior to stroke with/without continuous use after admission can reduce the risk of post-stroke pneumonia remains controversial because some studies support it (17, 18) while others do not (19, 20). The impact of statin before stroke on the risk of infection was investigated in many studies, whereas only a few studies gave attention to statin use in the acute phase. Moreover, statin treatment in the acute phase appeared to be associated with a higher post-stroke pneumonia risk (19, 21). Information on the potential effects of statins on post-stroke pneumonia severity in AIS patients is currently unavailable, while the prognosis of stroke patients might be affected by pneumonia severity.

Therefore, we conducted this retrospective study to investigate the impacts of statin on the risk and severity of post-stroke pneumonia in stroke patients during the acute phase.

## Materials and Methods

### Participants

The study recruited consecutive patients admitted to the Department of Neurology, West China Hospital, Sichuan University, within 72 h after stroke onset from January 2014 to February 2019. The inclusion criteria were as follows: (1) AIS diagnosed by the World Health Organization criteria on admission; (2) age ≥18 years; and (3) time from onset to admission ≤ 3 days. Patients were excluded if they present with: (1) a history of stroke or transient ischemic attack (TIA); (2) prophylactic antibiotic therapy before stroke or during hospitalization; (3) suffering from infectious or immunologic disease before stroke or receiving immunoglobulin therapies or immunosuppressive medications; (4) statin treatment during the non-acute phase (prior to stroke onset or at a later time point during the hospital stay); (5) pregnancy; and (6) post-stroke infections other than post-stroke pneumonia.

Ethical clearance of this study was provided by the Ethics Committee of West China Hospital. The procedures of this study adhere to the tenets of the Declaration of Helsinki. We registered the present study in the Chinese Clinical Trial Registry (ChiCTR 2000032838) on May 12, 2020.

### Data Collection

Information on patient gender, age, presence of dysphagia, blood chemistry test results [total cholesterol (TC), triglycerides (TG), high-density lipoprotein cholesterol (HDL-C), and low-density lipoprotein cholesterol (LDL-C)], stroke risk factors, and comorbidities [smoking status, hypertension, hypercholesterolemia, coronary arterial disease, atrial fibrillation, diabetes mellitus, and chronic obstructive pulmonary disease (COPD)] were recorded on admission. Data on stroke subtype, therapies during hospitalization, complications, blood chemistry examinations, medical interventions, and imaging (CT and/or MRI) results were obtained when discharged.

Stroke severity on admission was measured by the National Institutes of Health Stroke Scale (NIHSS). The AIS etiologic subtype was classified using the TOAST criteria (Trial of ORG 10172 in Acute Stroke Treatment). When stroke of probable atherosclerotic origin was considered, statins were used regardless of the LDL-C level (15). When statin was started within 3 days after stroke onset and lasted at least 3 days, it was defined as statin treatment in the acute phase, according to a previous study (22). We divided all eligible patients into the statin group with statin treatment in the acute phase and the non-statin group without any statin treatment. We used propensity score matching to adjust for differences in the baseline data of the two groups.

Standardized criteria of the US Centers for Disease Control and Prevention were used to diagnose post-stroke pneumonia. Apart from pulmonary infiltrates in chest X-rays, there must be at least one occurrence of the first and at least two of the second category: (1) fever (>38.0?C), leukocytosis (>12 × 10^9^/L) or leukopenia (<4 × 10^9^/L), and new inexplicable mental disorder in patients at least 70 years old; (2) new or worsening cough, labored breathing, polypnea, abnormality of respiratory tests, new or alterative purulent sputum, and damaged gas exchange (23).

### Outcomes

The patients were followed until hospital discharge. We considered risk of post-stroke pneumonia during the hospital stay as the primary outcome. The secondary outcome was the severity of post-stroke pneumonia, which was measured using the CURB-65 score during the hospital stay. The CURB-65 score (24) was assessed as follows: (1) confusion; (2) blood urea nitrogen above 7 mmol/L; (3) respiratory rate of 30 breaths per minute or more; (4) systolic blood pressure below 90 mmHg or diastolic blood pressure below 60 mmHg; and (5) aged 65 years and older.

### Propensity Matching

Propensity scores were acquired by using logistic regression analysis, with statin treatment in the acute phase as the dependent variable. A 1:1 matching range by using proximity matching was performed to match patients in the statin group to patients in the non-statin group with a caliper width of 0.01. The variables used for the propensity matching process were age, sex, the NIHSS score on admission, smoking status, hypertension, hypercholesterolemia, coronary arterial disease, atrial fibrillation, diabetes mellitus, dysphagia, tracheotomy/endotracheal intubation, nasogastric feeding tube, TC, TG, LDL-C, HDL-C, and the TOAST subtype.

### Data Analysis

Statistical analysis was conducted using SPSS 22.0 statistic software (SPSS Inc., Chicago, IL, USA) for Windows. Student's *t*-tests or Mann–Whitney tests were used to compare continuous variables, as appropriate. Chi-square tests and the Kruskal–Wallis tests were performed to compare categorical variables, as appropriate. Logistic regression analysis was used to analyze the association between the risk and severity of post-stroke pneumonia and statin treatment in the acute phase. The multivariate regression analysis included all variables with *p* <0.10 in univariate logistic regression and the covariates of interest in previous articles. The logistic regression model was analyzed using the enter method and the condition method before and after propensity matching, respectively. All tests were performed as two-sided tests, and differences were considered to indicate statistical significance at *p* < 0.05.

## Results

### Sample Population

**Figure 1:**
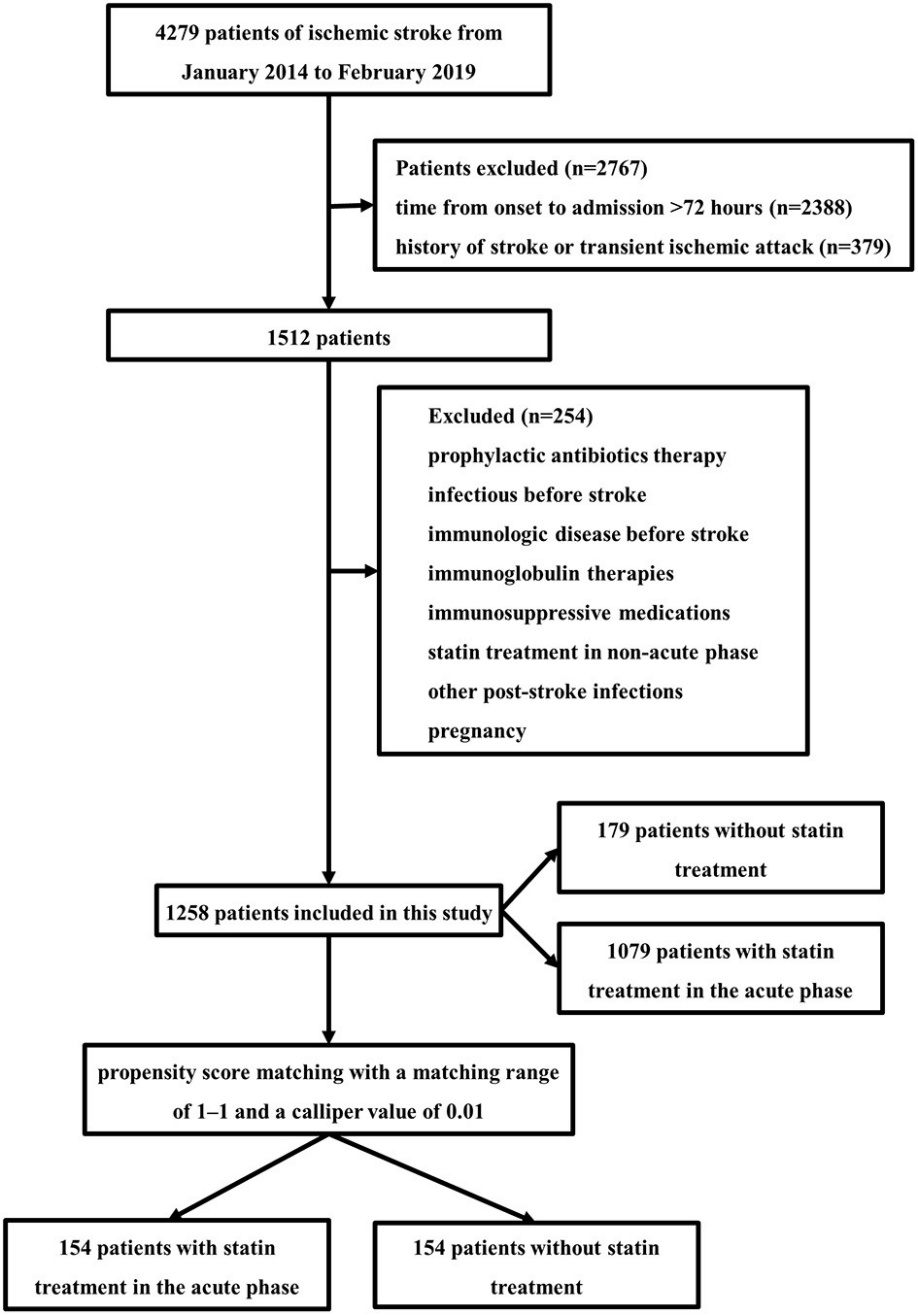
Flow diagram of included participants.

The study population comprised 1,258 AIS patients (Figure 1) with or without statin in the acute phase. In all patients, 746 were males (59.3%), with a mean age of 62.82 (± 14.34) years. A total of 1,079 (85.8%) patients received statin treatment in the acute phase, and 179 (14.2%) patients did not receive any statin treatment during the hospital stay. The majority of patients received atorvastatin (70.2%). Other statins included rosuvastatin (14.6%) and simvastatin. Atorvastatin was given at least 20 mg daily, simvastatin was given at least 40 mg daily, and rosuvastatin was given at least 10 mg daily.

Of the 1,258 patients, those with statin treatment in the acute phase were older and more likely to be men. The presence of hypertension, diabetes mellitus, hypercholesterolemia, smoking, and a stroke etiologic subtype of large artery atherosclerosis was more common in patients with statin treatment, whereas coronary artery disease, atrial fibrillation, dysphagia, and infection-related medical interventions (tracheotomy/endotracheal intubation and nasogastric feeding tube) were less common. Also, these patients had lower stroke severity and higher lipid levels on admission (TC and LDL-C) (Table 1).

**Table 1:** Baseline characteristics before and after propensity score matching.

**Variable**	**Total patients**	**Unmatched**			**Matched**		
	***N* = 1,258**	**Statin**	**Non-statin**	***P***		**Statin**	**Non-statin**	P^*^
		***N* = 1,079**	***N* = 179**			***N* = 154**	***N* = 154**	
Age^†^, years, mean (SD)	62.82 (14.34)	63.53 (13.75)	58.56 (16.88)	<0.001		58.01 (15.6)	58.64 (17.3)	0.74
Male^†^, %	746 (59.3)	664 (61.5)	82 (45.8)	<0.001		76 (49.4)	73 (47.4)	0.73
Hypertension^†^, %	702 (55.8)	642 (59.5)	60 (33.5)	<0.001		49 (31.8)	59 (38.3)	0.21
Diabetes mellitus^†^, %	292 (23.2)	268 (24.8)	24 (13.4)	<0.001		18 (11.7)	22 (14.3)	0.51
Hypercholesterolemia^†^, %	193 (15.3)	178 (16.5)	15 (8.4)	0.005		14 (9.1)	14 (9.1)	1.0
Coronary arterial disease^†^, %	120 (9.5)	89 (8.2)	31 (17.3)	<0.001		23 (14.9)	21 (13.6)	0.74
Atrial fibrillation^†^, %	263 (20.9)	205 (19.0)	58 (32.4)	<0.001		47 (30.5)	49 (31.8)	0.81
COPD, %	23 (1.8)	22 (2.0)	1(0.6)	0.17		3 (1.9)	1 (0.6)	0.34
Smoking^†^, %	355 (28.2)	322 (29.8)	33 (18.4)	0.002		35 (22.7)	31 (20.1)	0.56
TC^†^, mmol/L	4.12 (1.43)	4.24 (1.38)	3.37 (1.50)	<0.001		3.56 (1.41)	3.45 (1.40)	0.50
TG^†^, mmol/L	1.85 (1.38)	1.82 (1.34)	2.04 (1.56)	0.58		1.99 (1.57)	2.01 (1.57)	0.94
HDL-C^†^, mmol/L	1.32 (0.60)	1.31 (0.56)	1.38 (0.80)	0.14		1.37 (0.83)	1.38 (0.84)	0.89
LDL-C^†^, mmol/L	2.59 (1.06)	2.63 (0.96)	2.39 (1.52)	<0.001		2.33 (0.81)	2.31 (0.77)	0.76
NIHSS score on admission^†^, Med (IQR)	4 (2–10)	4 (2–9)	7 (2–13)	<0.001		5 (2–12)	7 (2–11)	0.56
Dysphagia^†^, %	126 (10.0)	92 (8.5)	34 (19.0)	<0.001		24 (15.6)	26 (16.9)	0.76
Tracheotomy/endotracheal intubation^†^, %	28 (2.2)	16 (1.5)	12 (6.7)	<0.001		8 (5.2)	7 (4.6)	0.80
Nasogastric feeding tube^†^, %	166 (13.2)	101 (9.4)	65 (36.3)	<0.001		42 (27.3)	44 (28.6)	0.78
TOAST subtypes^†^, %				<0.001				
LAA	630 (50.1)	583 (54.0)	47 (26.3)			52 (33.8)	46 (29.9)	0.51
CE	195 (15.5)	140 (13.0)	55 (30.7)			33 (21.4)	44 (28.6)	0.17
SVO	79 (6.3)	73 (6.8)	6 (3.4)			10 (6.5)	6 (3.9)	0.60
ODE	75 (6.0)	59 (5.5)	16 (8.9)			18 (11.7)	15 (9.7)	0.96
UDE	279 (22.2)	224 (20.8)	55 (30.7)			41 (26.6)	43 (27.9)	0.57
Length of hospital stay, Med (IQR)	10 (7–13)	10 (7–13)	10 (7–15)	0.77		10 (7–14)	10 (7–14)	0.16

*SD, standard distance; Med, median value; IQR, interquartile range; LAA, large-artery atherosclerosis; CE, cardioembolism; SVO, small-vessel occlusion; ODE, stroke of other determined cause; UDE, stroke of undetermined cause*.

† *Represents the propensity-matched variables*.

*P is calculated by t-test, Chi-square test, or Kruskal–Wallis test as appropriate*.

P* is calculated by conditional logistic regression.

### Primary Outcomes

The risk of post-stroke pneumonia was 21.5% (*n* = 271) during the hospital stay. Post-stroke pneumonia was less likely to occur in patients of the statin group before matching (19.4 *vs*. 34.6%, *p* < 0.001) (Table 2). Univariate logistic associations found that post-stroke pneumonia was associated with less use of statins in the acute phase (OR = 0.45, 95% CI = 0.32–0.64, *p* < 0.001) (Table 3). However, multivariate logistic analysis showed that the increased risk of post-stroke pneumonia was not significantly associated with statin in the acute phase (OR = 1.51, 95% CI = 0.85–2.67, *p* = 0.157) (Table 3). In addition, this outcome lost significance after propensity matching (35.1 *vs*. 27.9%, *p* = 0.155) (Table 2), and the association of statin with post-stroke pneumonia risk in the multivariate model was consistent with that before matching (OR = 1.57, 95% CI = 0.77–3.18, *p* = 0.213) (Table 3).

**Table 2:** Outcome measures of unmatched and propensity-matched subgroups.

**variable**	**Unmatched**			**Matched**		
	**Statin**	**Non-statin**	***P***	**Statin**	**Non-statin**	P^*^
Post-stroke pneumonia, %	209 (19.4)	62 (34.6)	<0.001	54 (35.1)	43 (27.9)	0.16
Pneumonia severity, Med (IQR)	2 (1–2)	2 (1–2)	0.17	—	—	—
Pneumonia severity (CURB-65 3–5), %	41 (19.6)	12 (19.4)	0.96	—	—	—

*Med, median value; IQR, interquartile range. P is calculated by t-test, Chi-square test, or Kruskal–Wallis test as appropriate*.

P* is calculated by conditional logistic regression.

**Table 3:** Univariate and multivariate analysis for the risk of post-stroke pneumonia before and after propensity score matching.

**Variable**	**Unmatched**				**Matched**			
	**Univariate**	***P***	**Multivariate**	P*	**Univariate**	***P***	**Multivariate**	P*
	**β (95% CI)**		**β (95% CI)**		**β (95% CI)**		**β (95% CI)**	
Age	1.03 (1.02–1.04)	<0.001	1.01 (0.99–1.02)	0.25	1.03 (1.02–1.05)	<0.001	1.01 (0.99–1.04)	0.30
Male	0.81 (0.62–1.07)	0.14	0.95 (0.62–1.45)	0.82	1.53 (0.95–2.49)	0.083	1.52 (0.70–3.27)	0.29
Hypertension	0.79 (0.61–1.04)	0.092	1.04 (0.70–1.53)	0.86	1.07 (0.65–1.76)	0.80	—	—
Diabetes mellitus	0.36 (0.24–0.54)	<0.001	0.40 (0.25–0.67)	<0.001	0.42 (0.18–0.99)	0.046	0.16 (0.04–0.56)	0.004
Hypercholesterolemia	0.63 (0.42–0.95)	0.029	0.91 (0.50–1.64)	0.74	1.03 (0.45–2.38)	0.94	—	—
Coronary arterial disease	6.53 (4.40–9.67)	<0.001	4.07 (2.35–7.05)	<0.001	8.16 (3.97–16.78)	<0.001	9.81 (3.31–29.09)	<0.001
Atrial fibrillation	2.82 (2.09–3.81)	<0.001	1.31 (0.82–2.08)	0.26	1.13 (0.68–1.89)	0.64	—	—
COPD	1.29 (0.50–3.31)	0.59	0.53 (0.13–2.17)	0.38	2.20 (0.31–15.85)	0.43	0.30 (0.02–3.68)	0.35
Smoking	1.14 (0.85–1.52)	0.40	1.38 (0.88–2.16)	0.16	1.70 (0.97–2.99)	0.065	2.47 (0.99–6.14)	0.052
TC	0.81 (0.74–0.90)	<0.001	1.00 (0.83–1.21)	0.99	0.95 (0.80–1.13)	0.58	—	—
LDL-C	0.69 (0.59–0.81)	<0.001	0.95 (0.72–1.25)	0.71	0.76 (0.55–1.04)	0.088	0.84 (0.53–1.32)	0.44
NIHSS score on admission	1.17 (1.14–1.20)	<0.001	1.07 (1.03–1.10)	<0.001	1.14 (1.10–1.19)	<0.001	1.03 (0.97–1.09)	0.36
Dysphagia	8.57 (5.77–12.71)	<0.001	2.59 (1.48–4.53)	0.001	6.58 (3.40–12.71)	<0.001	2.21 (0.83–5.87)	0.11
Tracheotomy/ endotracheal intubation	23.88 (8.21–69.45)	<0.001	4.52 (1.18–17.40)	0.028	6.62 (2.05–21.36)	0.002	3.87 (0.58–26.10)	0.16
Nasogastric feeding tube	20.57 (13.84–30.58)	<0.001	7.29 (4.43–12.01)	<0.001	15.15 (8.30–27.63)	<0.001	8.26 (3.60–18.94)	<0.001
**TOAST subtypes**								
LAA	ref	Ref	Ref	Ref	Ref	Ref	Ref	Ref
CE	2.48 (1.74–3.54)	<0.001	1.12 (0.65–1.92)	0.69	0.96 (0.51–1.80)	0.90	0.61 (0.24–1.59)	0.31
SVO	0.23 (0.08–0.65)	0.005	0.58 (0.20–1.71)	0.32	0.27 (0.06–1.25)	0.094	1.39 (0.23–8.33)	0.72
ODE	1.09 (0.60–1.98)	0.79	1.31 (0.59–2.88)	0.51	0.82 (0.35–1.92)	0.64	1.54 (0.48–4.98)	0.47
UDE	1.27 (0.90–1.79)	0.18	1.31 (0.81–2.12)	0.27	0.80 (0.43–1.49)	0.48	1.80 (0.71–4.61)	0.22
Statin	0.45 (0.32–0.64)	<0.001	1.51 (0.85–2.67)	0.16	1.39 (0.86–2.26)	0.18	1.57 (0.77–3.18)	0.21
Length of hospital stay	1.16 (1.13–1.18)	<0.001	1.12 (1.09–1.15)	<0.001	1.13 (1.08–1.18)	<0.001	1.14 (1.07–1.21)	<0.001

*LAA, large-artery atherosclerosis; CE, cardioembolism; SVO, small-vessel occlusion; ODE, stroke of other determined cause; UDE, stroke of undetermined cause*.

P^*^ value was analyzed by the enter method of logistic regression analysis.

### Secondary Outcome

Of the 271 (21.5%) patients who presented with post-stroke pneumonia, we found no significant differences of post-stroke pneumonia severity during hospital stay between patients treated with statin in the acute phase and patients without statin treatment (19.6 *vs*. 19.4%, *p* = 0.964) (Table 2). We did not find statin use significantly reducing the severity of post-stroke pneumonia in the univariate logistic analysis (OR = 1.02, 95% CI = 0.50–2.08, *p* = 0.964) (Table 4). Moreover, multivariate logistic analysis showed that statin treatment in the acute phase did not significantly influence the severity of post-stroke pneumonia (OR = 0.95, 95% CI = 0.39–2.31, *p* = 0.918) (Table 4).

**Table 4:** Univariate and multivariate logistic regression analysis for severe post-stroke pneumonia (CURB-65 score ≥ 3).

**Variable**	**Univariate**	***P*-value**	**Multivariate**	P* value
	**β (95% CI)**		**β (95% CI)**	
Age^‡^	1.09 (1.05–1.12)	<0.001	1.09 (1.05–1.13)	<0.001
Male^‡^	0.73 (0.40–1.33)	0.31	0.92 (0.45–1.88)	0.83
Hypertension	0.90 (0.49–1.63)	0.72	—	—
Diabetes mellitus	0.77 (0.28–2.11)	0.61	—	—
Hypercholesterolemia	0.80 (0.29–2.21)	0.67	—	—
Coronary arterial disease	1.64 (0.86–3.14)	0.13	—	—
Atrial fibrillation^‡^	1.77 (0.97–3.26)	0.065	1.29 (0.65–2.56)	0.47
COPD	2.10 (0.37–11.77)	0.40	—	—
Smoking	0.70 (0.35–1.40)	0.31	—	—
NIHSS score on admission^‡^	1.08 (1.04–1.12)	<0.001	1.08 (1.03–1.12)	0.001
Dysphagia	0.44 (0.05–3.49)	0.43	—	—
Tracheotomy/ endotracheal intubation^‡^	2.24 (0.91–5.57)	0.081	1.44 (0.48–4.37)	0.52
Nasogastric feeding tube^‡^	2.45 (1.32–4.57)	0.005	1.75 (0.83–3.69)	0.14
TOAST subtypes
LAA	Ref	Ref	—	—
CE	0.93 (0.44–1.97)	0.84	—	—
SVO	0.00 (0.00–Inf)	0.98	—	—
ODE	0.64 (0.13–3.01)	0.57	—	—
UDE	1.29 (0.62–2.70)	0.50	—	—
Statin^‡^	1.02(0.50–2.08)	0.96	0.95 (0.39–2.31)	0.92
Length of hospital stay	1.03 (0.99–1.06)	0.12	—	—

*LAA, large-artery atherosclerosis; CE, cardioembolism; SVO, small-vessel occlusion; ODE, stroke of other determined cause; UDE, stroke of undetermined cause*.

*Dependent variable: post-stroke pneumonia severity (CURB-65) 0–2 vs. 3–5*.

‡ *Values were analyzed in the multivariable model*.

P^*^ value was analyzed by the enter method of logistic regression analysis.

## Discussion

In terms of the risk of post-stroke pneumonia, we did not find significant differences between patients with statin treatment in the acute phase and those without any statin treatment when they were adjusted, suggesting its potent immunomodulatory properties on post-stroke pneumonia (25). In addition, no significant association was observed between acute statin treatment and the severity of post-stroke pneumonia in this study.

We did not find a significant association between statin in the acute phase and the increased risk of post-stroke pneumonia in the multivariate model, and the propensity score matching analysis confirmed this result. Our findings are consistent with the results observed by Becker et al. in a retrospective study of 112 ischemic stroke patients, where the risk of post-stroke infections was increased in patients using statins at the time of onset or initiating within the first 3 days after onset compared to those using statins later or not at all, but the difference was not statistically significant (19). Our results differed from several published studies (17, 18) that found statin treatment could reduce the risk of post-stroke infections among AIS patients with thrombolysis or without endotracheal intubation. This inconsistency was mainly explained by differences in the study design, such as the criteria for eligible patients, post-stroke infection subtypes, and the classification of statin initiation and duration.

The research result that statins did not appear to influence the risk of post-stroke pneumonia may be most plausibly explained by their potent immunomodulatory properties, although the multiple potential pharmacological mechanisms of statins on post-stroke infection remain unclear. On the one hand, statins could disturb the activation of the body's innate immunity process (26). Statins are related to the inhibition of the expression of major histocompatibility complex II, and they suppress the immune responses mediated by T helper lymphocyte type 1. Additionally, statins could reduce the secretion of proinflammatory cytokines (11, 27–29). On the other hand, statins suppress the acquired immunity process by inhibiting antigen presentation and reducing the T cell function and proliferation (30).

A surprising finding was that acute statin treatment was not significantly associated with the severity of post-stroke pneumonia. Our results might be explained by post-stroke pneumonia patients having more severe conditions and poorer prognosis compared to patients without post-stroke pneumonia, and the effects of statins might be so weak that these were obscured by the severe conditions. Moreover, several confounders that were not included or not fully controlled might have resulted in the negative findings. Therefore, the definite effect of statins on the severity of post-stroke pneumonia remains to be elucidated and requires further investigation.

To our knowledge, this is the first population-based study to establish a relationship between severity of post-stroke pneumonia and statin treatment in the acute phase, although in the end we found no significant differences between them. In order to eliminate the influence of the differences in statin treatment in patients with a history of stroke, our study only enrolled first-time ischemic stroke patients. Furthermore, we clearly administered statin treatment only in the acute phase, and we adjusted for some important infection-related medical interventions in the statistical analysis. Lastly, propensity score matching was performed to further control for the influence of confounding factors.

Several limitations must be considered in interpreting our findings. Firstly, there are unavoidable information and selection bias in this retrospective study. The results need to be interpreted with caution, and additional studies are required. Secondly, the results were confined to patients who were admitted to hospital within 72 h after stroke onset. Thirdly, despite the matching and adjustment, not all factors could be captured and residual confounding would still remain. Finally, we did not stratify the effects of each category of statins at different doses because satins, with the exception of atorvastatin, were rarely used in our study. Given that some statins had clear advantages over others in decreasing the morbidity and mortality of infection when administered pre-infection (31), studying the effects of different satins might be significant. In addition, the dose of statins has been shown to affect neuroprotective effects in several experimental studies (32–34). Therefore, further research with a large group of patients and adequate information to allow for powerful stratification analysis is required to confirm the results.

## Conclusion

In summary, the results of the present study provide proof that statin treatment in the acute phase might not appear to increase the risk and severity of post-stroke pneumonia. There were no additional benefits of statin treatment in the acute phase for post-stroke pneumonia reduction (including risk and severity) among AIS patients. Further observational studies or clinical trials with large databases are required to confirm our results.

## Data Availability Statement

The original contributions presented in the study are included in the article/supplementary material, further inquiries can be directed to the corresponding author/s.

## Ethics Statement

The studies involving human participants were reviewed and approved by The Ethics Committee of West China Hospital. Written informed consent for participation was not required for this study in accordance with the national legislation and the institutional requirements.

## Author Contributions

CL and MM were responsible for data analysis and manuscript drafting and revision. SD and YH revised the manuscript. JB, YZ, LG, and CC were responsible for data collection and follow-up. JG and LH designed the research. All authors reviewed the successive versions of the manuscript and approved the final version.

## Conflict of Interest

The authors declare that the research was conducted in the absence of any commercial or financial relationships that could be construed as a potential conflict of interest.

## Publisher's Note

All claims expressed in this article are solely those of the authors and do not necessarily represent those of their affiliated organizations, or those of the publisher, the editors and the reviewers. Any product that may be evaluated in this article, or claim that may be made by its manufacturer, is not guaranteed or endorsed by the publisher.

## Abbreviations

AIS: acute ischemic stroke
PSI: post-stroke infections
TIA: transient ischemic attack
ChiCTR: Chinese Clinical Trial Registry
TC: total cholesterol
LDL-C: low-density lipoprotein cholesterol
HDL-C: high-density lipoprotein cholesterol
TG: triglycerides
COPD: chronic obstructive pulmonary disease
NIHSS: National Institutes of Health Stroke Scale
TOAST: Trial of ORG 10172 in Acute Stroke Treatment.
